# Alignment of Alzheimer’s disease amyloid β-peptide and klotho

**DOI:** 10.3892/wasj.2020.68

**Published:** 2020-09-22

**Authors:** STEVEN LEHRER, PETER H. RHEINSTEIN

**Affiliations:** 1 Department of Radiation Oncology, Icahn School of Medicine at Mount Sinai, New York, NY 10029;; 2 Severn Health Solutions, Severna Park, MD 21146, USA

**Keywords:** β-amyloid, Alzheimer’s disease, protein, alignment, klotho, aging, neurodegeneration, HSV-1, neuroinflammation, ubiquitin

## Abstract

The cause of Alzheimer’s disease (AD) is poorly understood. In 1991, the amyloid hypothesis postulated that β-amyloid (Aβ) accumulation is a key element. It follows that clearing the brain of Aβ would be beneficial, which has not been the case. Therefore, Aβ is likely a result, not a cause, of AD and may be protective rather than harmful. The apolipoprotein E4 (apoE4) allele is the strongest genetic risk factor for AD. Klotho (KL), encoded by the KL gene, may be another AD-related protein. FGF21 is a circulating endocrine hormone, mainly secreted by the liver, mostly during fasting. FGF21 acts by binding to its receptor FGFR1 and co-receptor β-klotho. FGF21 is neuroprotective and could delay onset of AD. In the present study, the KL protein structure was examined to determine whether it may interact with Aβ. Protein data bank (pdb) entries for klotho and Aβ were searched on the RCSB Protein Data Bank for β-KL and AD amyloid β-peptide. The protein structures were superimposed and aligned on PYMOL v2.3.4 with the super command, which super aligns two protein selections. To evaluate the conservation and alignment of the Aβ and KL genomes across species, BLAT, the Blast-Like Alignment Tool of the UCSC Genome Browser, was used. The amino acid residues phe76-val96 of KL aligned closely with residues asp7-asn27 of Aβ. Cross-species comparison of KL revealed a high degree of alignment and conservation in the chimp and 27 other primates; however, less alignment and conservation were observed in the mouse, dog and elephant, even less in the chicken, western clawed frog (*Xenopus tropicalis*), zebrafish and lamprey. The current finding of amino acid residues phe76-val96 of klotho aligning closely with residues asp7-asn27 of Aβ suggests that Aβ can enhance the ability of klotho to draw FGF21 to regions of incipient neurodegeneration in AD. The problem arises with age. Older individuals do not heal or repair tissue damage as well as younger individuals. As neurodegeneration advances in an older individual, perhaps caused by neuroinflammation related to herpes simplex virus type 1, increasing amounts of amyloid are produced, forming an adhesive web, as the brain tries to hold the pathologic process in check. Meanwhile, the damage increases and spreads. Progressive neurodegeneration and cognitive decline are the outcome.

## Introduction

The cause of Alzheimer’s disease (AD) is poorly understood. The disease process is associated with β-amyloid (Aβ) plaques, tau neurofibrillary tangles and neuroinflammation. In 1991, the amyloid hypothesis postulated that beta amyloid (Aβ) accumulation is a key element (1). Aβ was supposed to stimulate both the development of tau neurofibrillary tangles and neuroinflammation. Aβ, tau and inflammation each led to the destruction of neurons and synapses. It follows that clearing the brain of Aβ would be beneficial, which has not been the case. Therefore, Aβ is likely a result, not a cause, of AD (2,3) and may be protective rather than harmful (4).

Aβ has antimicrobial properties (4) and could represent a brain defense against infection (5), in particular against herpes simplex virus 1 (HSV-1). HSV-1 is found in regions of the brain that are affected by AD in elderly individuals. Additionally, neuronal infection with HSV-1 triggers the accumulation of amyloid beta deposits and hyperphosphorylated tau, and results in oxidative stress and synaptic dysfunction. These factors are implicated in the development of AD (6).

The apolipoprotein E4 (apoE4) allele is the strongest genetic risk factor for AD. Approximately 23% of the US population carries an apoE4 allele. The apoE2 allele is less common, 5% incidence, and is protective against AD.

Klotho (KL), encoded by the KL gene, may be another AD-related protein. In mice, elevated KL levels extend lifespan, enhance synaptic function and improve cognition during aging (7). Cognitively normal older individuals who have higher serum KL levels exhibit enhanced functional connectivity among brain regions that degenerate in AD (8).

Approximately 20% of individuals carry a KL variant, KL-VS. Heterozygosity (one copy) of KL-VS increases circulating klotho, while reducing Aβ and lowering AD risk in apoE4 carriers who are age 60 to 80 and cognitively normal (9). In the present study, the KL protein structure was examined to determine whether it may interact with Aβ.

## Data collection methods

**Figure 1.**
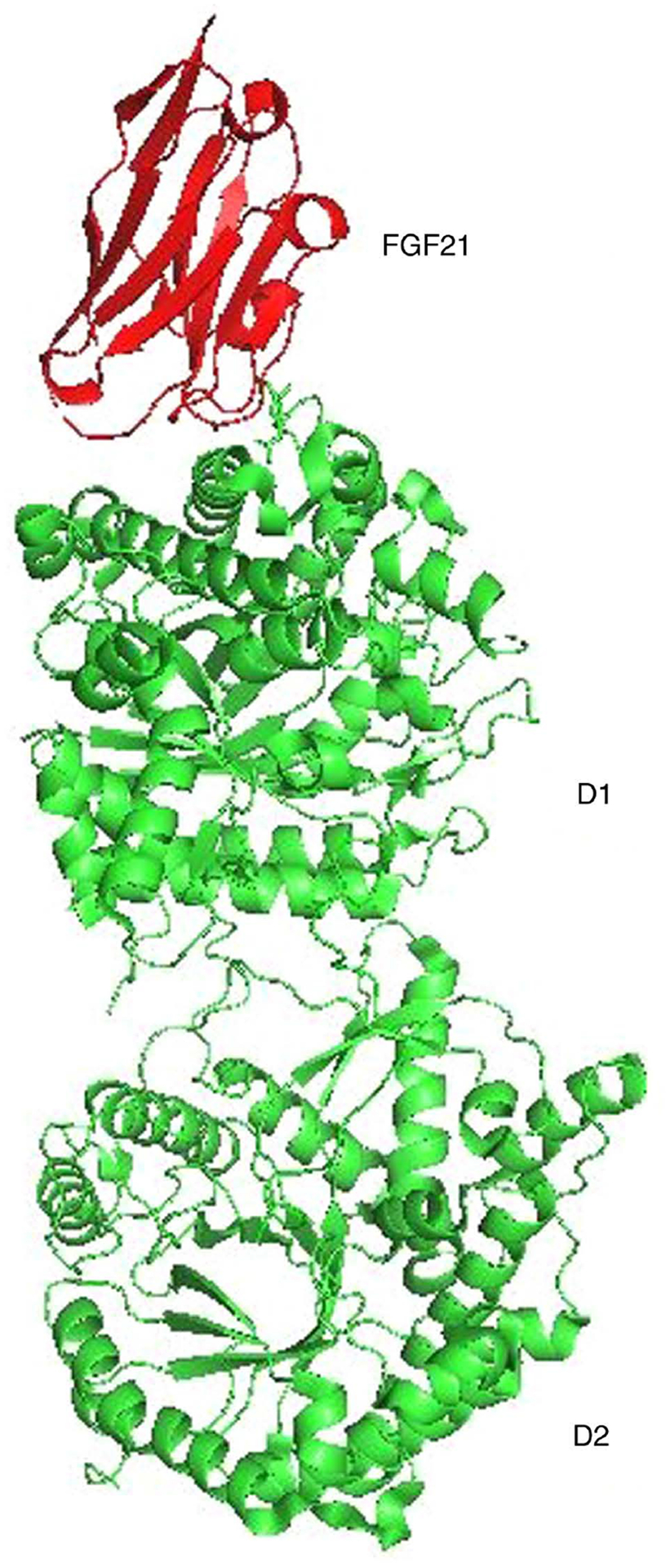
Crystal structure of β-Klotho showing FGF21 (red) bound to domain 1 (D1).

**Figure 2.**
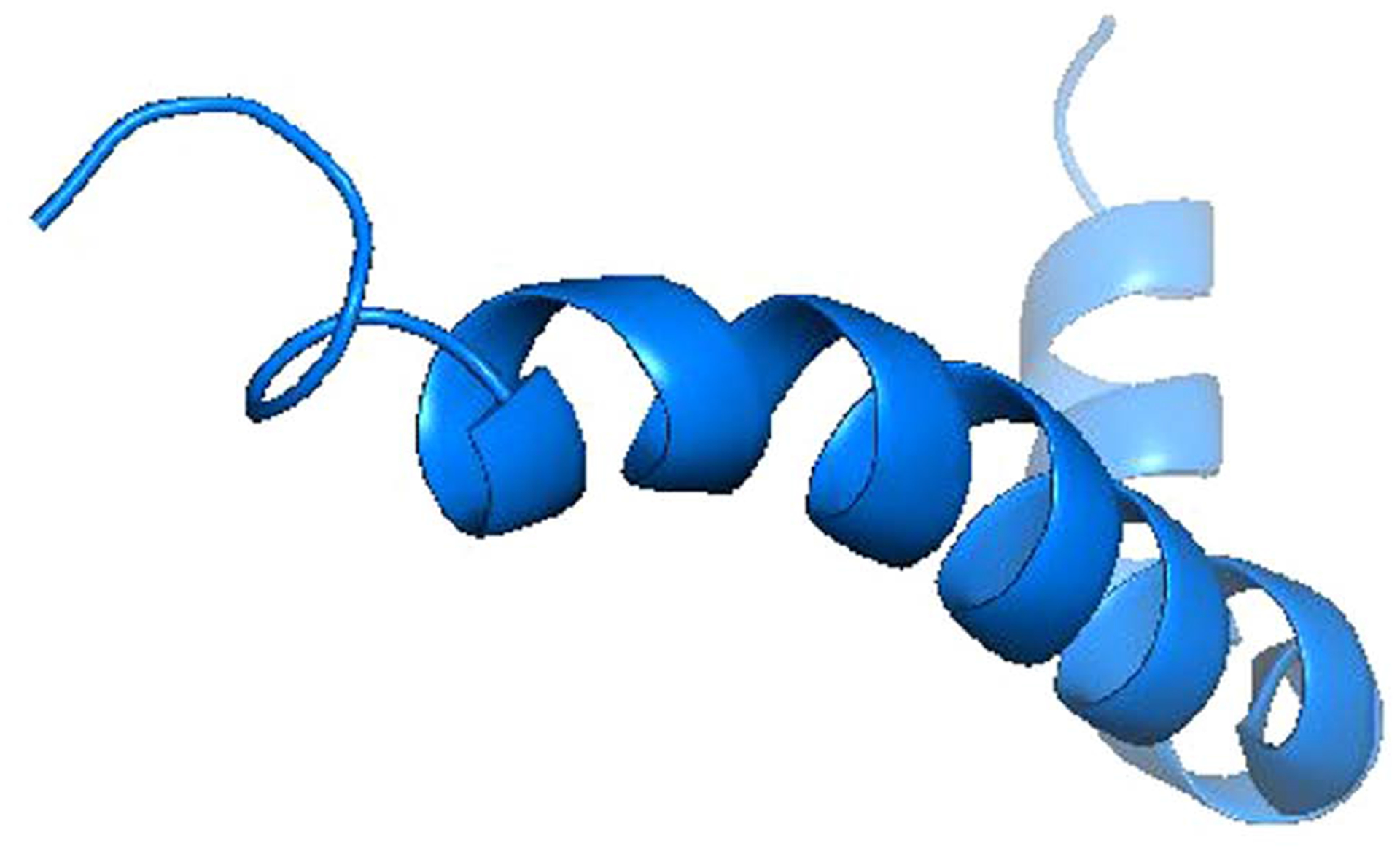
Solution structure of the Alzheimer’s disease Aβ-peptide.

Protein data bank (pdb) entries for KL and Aβ were searched on the RCSB Protein Data Bank. The following structures were identified: i) 5VAK (Fig. 1), representing the crystal structure of β-KL in complex with FGF21CT (C terminal tail). The method used was X-RAY diffraction. The resolution was 2.61 Å, structure deposited on March 27, 2017 and released on January 31, 2018 (10). ii) 1IYT (Fig. 2), representing the solution structure of the AD Aβ-peptide. The method used was solution NMR, structure deposited on September 6, 2002 and released on February 11, 2003 (11).

The protein structures were superimposed and aligned on PYMOL v 2.3.4 with the *Super* command, which super aligns two protein selections. *Super* does a sequence-independent structure-based dynamic programming alignment (unlike the *align* command) followed by a series of refinement cycles intended to improve the fit by eliminating pairing with high relative variability. The *Super* command is more reliable than *align* for proteins with low sequence similarity.

To evaluate conservation and alignment of the Aβ and KL genomes across species, we used BLAT, the Blast-Like Alignment Tool of the UCSC Genome Browser (12). BLAT can align a user sequence of 25 bases or more to the genome. As some level of mismatch is tolerated, cross-species alignments may be performed provided the species have not diverged too far from each other; this capability allowed comparison of the Mouse Mammary Tumor Virus genome to the human genome (13). BLAT calculates a percent identity score to indicate differences between sequences without a perfect match (i.e., without 100% identity). The differences include mismatches and gaps (14).

## Results

**Figure 3.**
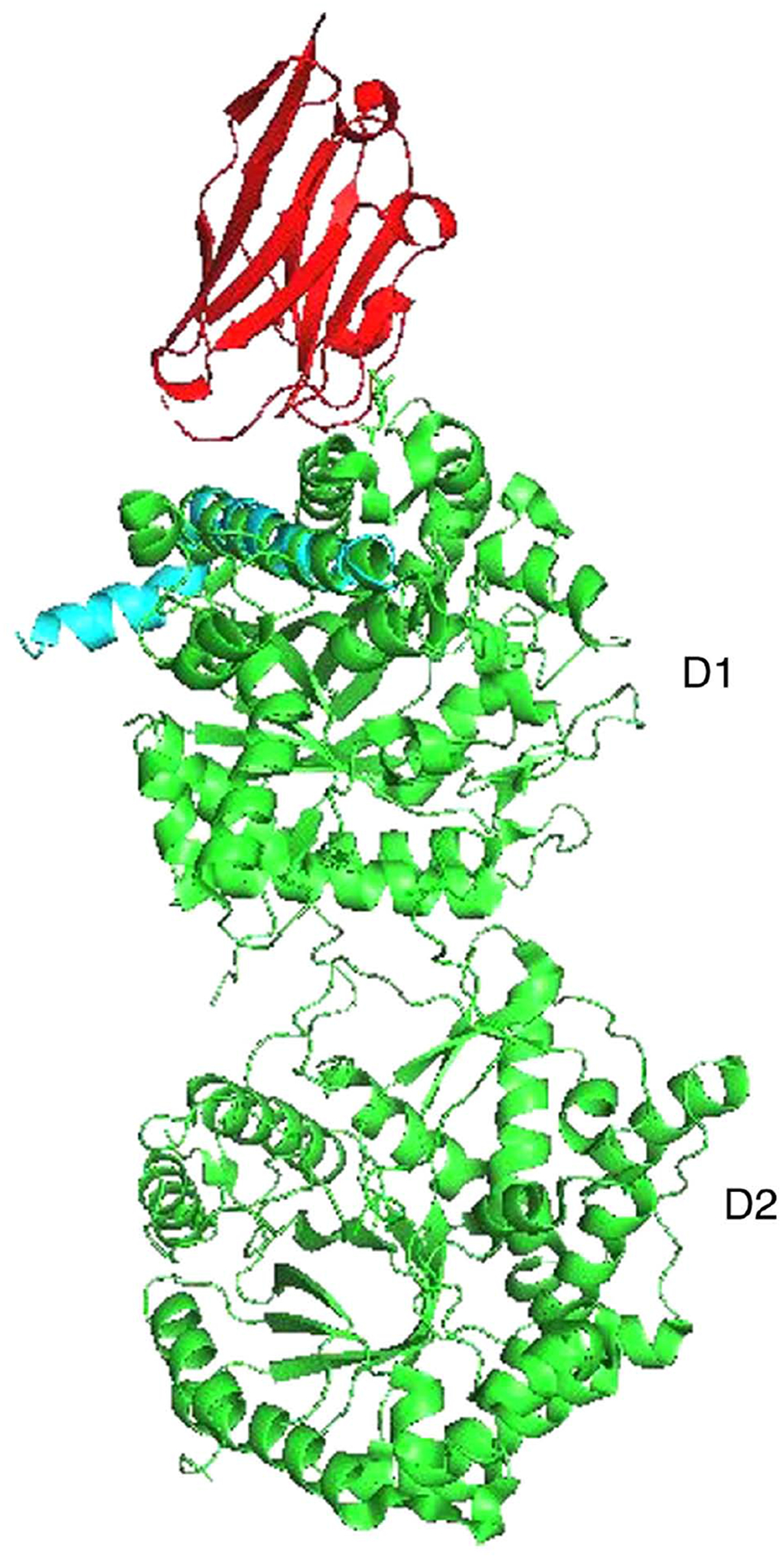
Alignment of klotho peptide (green) domain 1 (D1) with Aβ (blue). FGF21 (red) is bound to domain 1.

**Figure 4.**
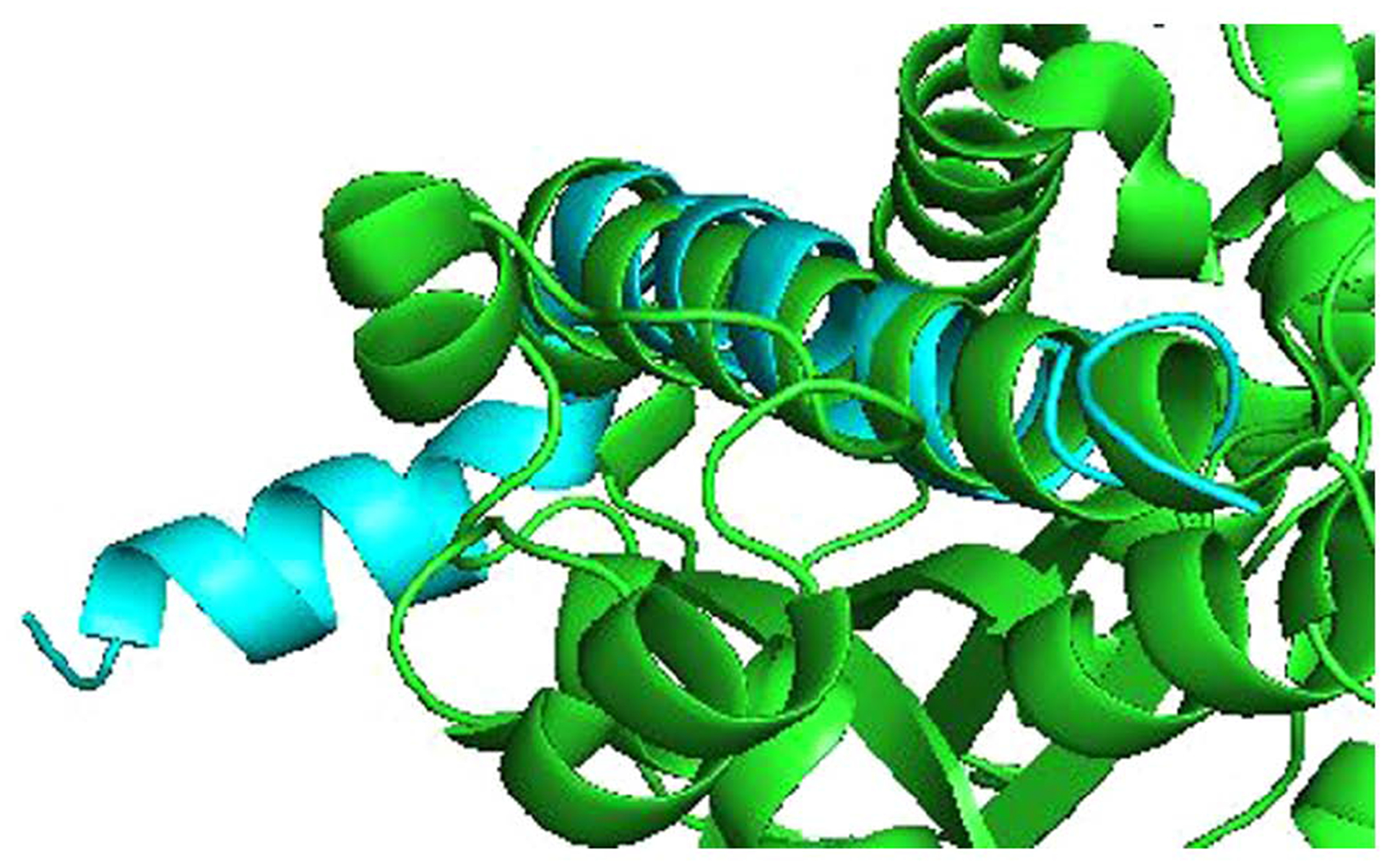
Closeup of alignment. Pymol performed 6 cycles of calculations on 165 aligned atoms, with a final root mean square deviation of atomic positions (RMSD) of 1.792 Å for 148 atoms. Amino acid residues phe76-val96 of klotho aligned closely with residues asp7-asn27 of Aβ.

For KL and Aβ, Pymol performed 6 cycles of calculations on 165 aligned atoms, with a final root mean square deviation of atomic positions (RMSD) of 1.792 Å for 148 atoms. Amino acid residues phe76-val96 of KL aligned closely with residues asp7-asn27 of Aβ (Figs. 3 and 4).

**Figure 5.**
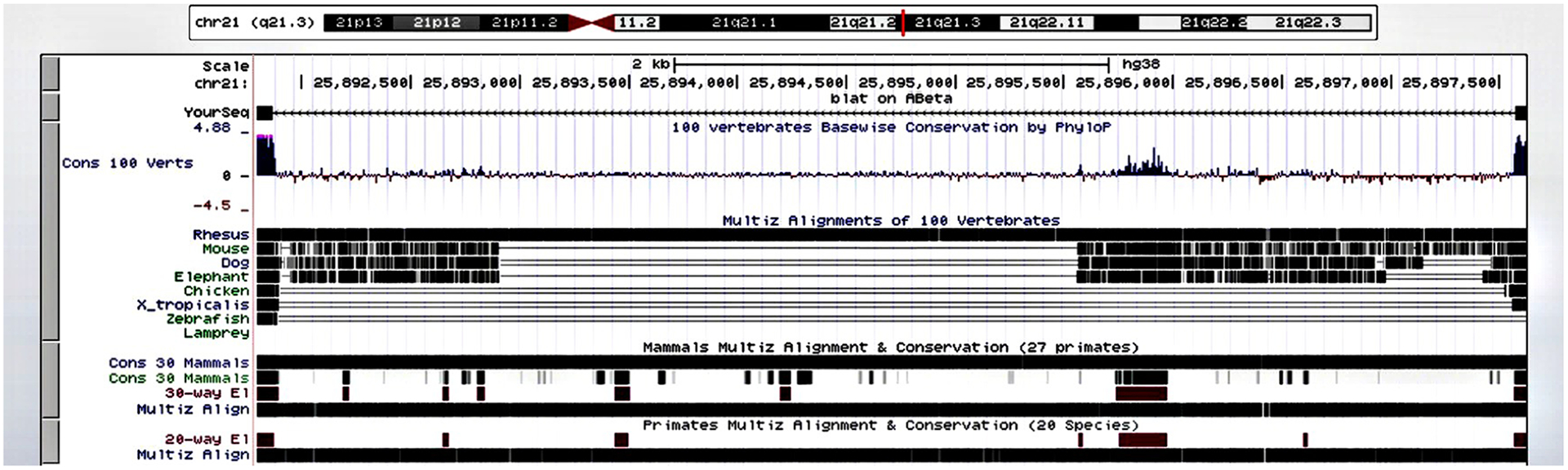
Alignment of 42 amino acid residue human Aβ across species in the UCSC genome browser. There is a high degree of alignment and conservation of Aβ (chr 21q21.3) in the rhesus monkey and 27 other primates, but much less alignment and conservation in the mouse, dog, and elephant, even less in the chicken, western clawed frog (*Xenopus tropicalis*), zebrafish and lamprey.

The results of the cross-species comparison of Aβ revealed a high degree of alignment and conservation of human Aβ (chr 21q21.3) in the rhesus monkey and 27 other primates. The rhesus macaque diverged from ancestors of *Homo sapiens* approximately 25 million years ago (15). There was much less alignment and conservation in the mouse, dog, and elephant, even less in the chicken, western clawed frog (*Xenopus tropicalis*), zebrafish and lamprey (Fig. 5).

**Figure 6.**
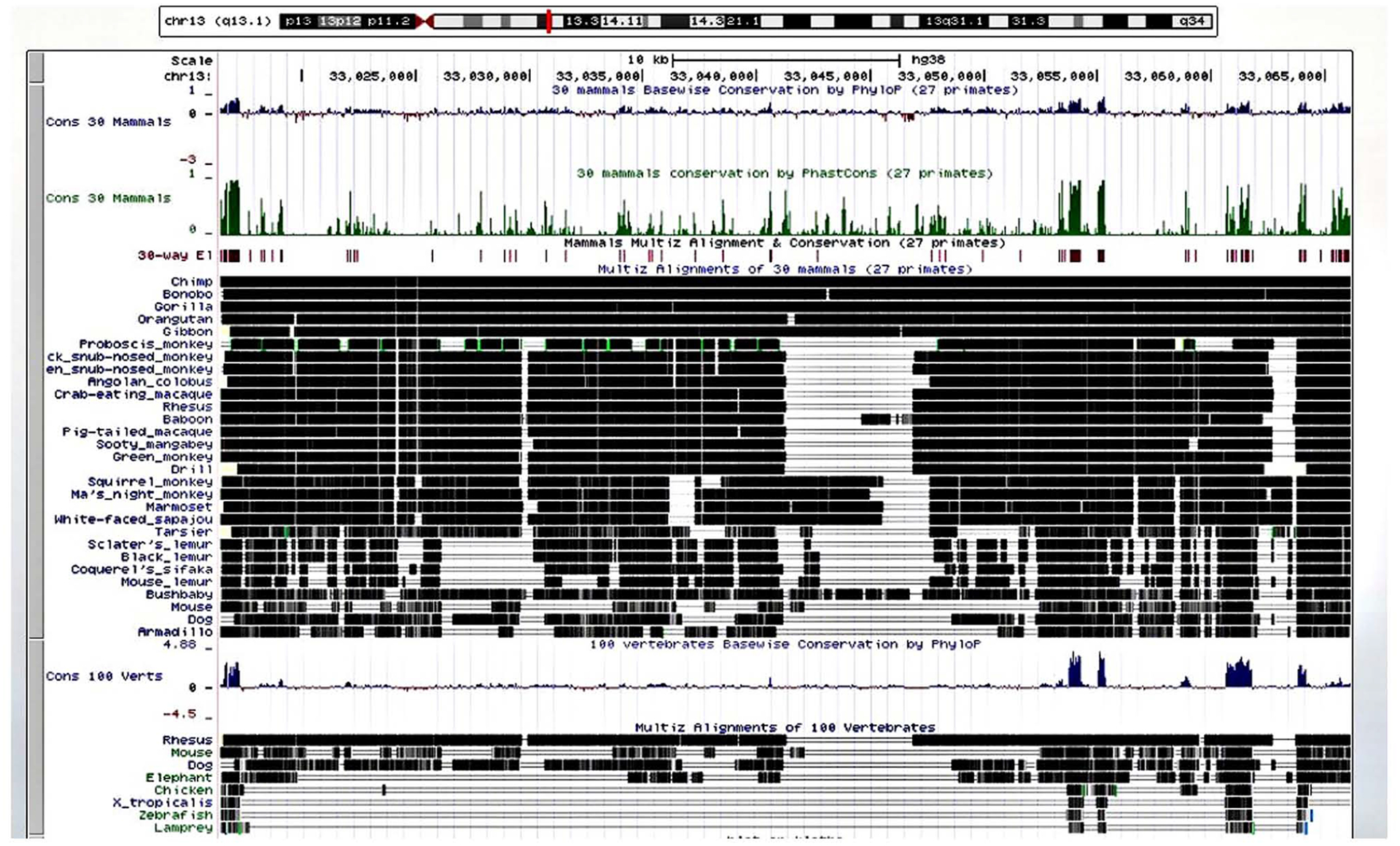
Alignment of human klotho across species in the UCSC genome browser. Results of the cross-species comparison of klotho show a high degree of alignment and conservation of human klotho (chr 13q13.1) in the chimp and 27 other primates, less alignment and conservation in the mouse, dog, and elephant, even less in the chicken, western clawed frog (*Xenopus tropicalis*), zebrafish and lamprey.

The results of the cross-species comparison of KL revealed a high degree of alignment and conservation of KL (chr 13q13.1) in the chimp and 27 other primates, with less alignment and conservation in the mouse, dog and elephant, even less in the chicken, western clawed frog (*Xenopus tropicalis*), zebrafish and lamprey (Fig. 6).

## Discussion

Aβ is an ancient neuropeptide expressed in vertebrates. Many primate species share the human Aβ sequence, which has been highly conserved over millions of years (16). The conservation of KL is similar. The high degree of conservation suggests that both sequences play an important role in survival.

KL is a membrane protein that is related to β-glucuronidases, enzymes that break down carbohydrates. KL has tandem glucosidase domains, D1 and D2 (Fig. 1). Three subtypes of KL have been identified: α-KL, β-KL and γ-KL. Low levels of KL are present in patients with chronic renal failure. KL may be one element involved in degenerative processes, such as arteriosclerosis, osteoporosis and skin atrophy often observed in renal failure. Mutations in the KL protein have been associated with aging, bone loss and alcohol consumption (17,18).

*In vivo*, Aβ and KL may function like ubiquitin and the substrate proteins to which it binds. Ubiquitin is a small (8.6 kDa) regulatory protein present in most tissues of eukaryotic organisms, that is, it occurs ubiquitously (19). The conjugation and binding of ubiquitin to a substrate protein is called ubiquitination. Ubiquitination affects proteins in many ways. Ubiquitin can alter protein cellular location, affect protein activity, and promote protein interactions. Similarly, after Aβ conjugates and binds to klotho, Aβ and KL could enhance the marking of brain regions for delivery of fibroblast growth factor 21 (FGF21) (10).

FGF21 is neuroprotective and may delay the onset of AD. FGF21 is a circulating endocrine hormone, mainly secreted by the liver, mostly during fasting. FGF21 acts by binding to its receptor FGFR1 and co-receptor β-KL. FGF21 regulates energy consumption by influencing glucose and lipid metabolism. Deranged FGF21 signaling might account for some forms of neurodegeneration, and FGF21 could be therapeutic in AD (20). Structurally, FGF21 is a 181 amino acid peptide (~22.3 kDa molecular mass) derived from a 209 amino acid mature protein encoded by the FGF21 gene located on chromosome 19.

ApoE4 is an independent risk factor for AD. KL can act on ApoE4 to prevent pathological β-amyloid production or deposition, enhance synaptic functions, and increased brain connectivity. The KL-VS status could thus mitigate ApoE4 risks for AD and could be used to further stratify individuals who carry APOE4 in clinical trials for the disease. KL itself could represent a therapeutic for the prevention or treatment of AD in individuals who carry ApoE4 (7). It would be vital in future molecular studies to factor in the relationship of ApoE4, klotho, and the occurrence of AD.

The present demonstration of amino acid residues phe76-val96 of KL aligning closely with residues asp7-asn27 of Aβ suggests that Aβ could enhance the ability of KL to draw FGF21 to regions of incipient neurodegeneration in AD. The problem arises with age. Older people do not heal or repair tissue damage as well as younger individuals. As neurodegeneration advances in an older individual, perhaps caused by neuroinflammation related to HSV-1 (21), increasing amounts of amyloid are produced, forming an adhesive web, as the brain tries to hold the pathologic process in check. Meanwhile, damage increases and spreads. Progressive neurodegeneration and cognitive decline are the outcome.

Further studies are required to explore these findings in depth. It would be worthwhile to examine the function of klotho and FGF21 in animal experiments.
